# Supplementary material for: Development and external validation of multivariate prediction models for erectile dysfunction in men with localized prostate cancer
Source: PLoS One. 2023 Mar 3;18(3):e0276815. doi: 10.1371/journal.pone.0276815 (PMC9983834; doi:10.1371/journal.pone.0276815)
Supplement: S1 Table — (PDF) [file pone.0276815.s001.pdf]

The variables used as input for the prediction models for both years. The models in which they were used are depicted as well as the question of the EPIC questionnaire they apply to (if applicable). Additionally, the possible input values are described with a number and the meaning of that number as input. The numbers in the last column are numbers from the data.

Variable	Input for		Relates to EPIC-26 question	Input values
	1y	2y		
Tumor T stage	x	x		1 = T1<br>2 = T2<br>3 = T3
Alcohol use	x			1 = no<br>2 = previously<br>3 = yes
Lack of energy	x		13d	1 = no problem<br>2 = very small problem<br>3 = small problem<br>4 = moderate problem<br>5 = big problem
The presence of cardiovascular disease (CVD)	x			1 = no CVD<br>2 = CVD
frequency of erections	x	x	10	1 = never when I wanted one<br>2 = <50% when I wanted one<br>3 = 50% when I wanted one<br>4 = >50% when I wanted one<br>5 = whenever I wanted one
quality of erections	x	x	9	1 = none at all<br>2 = not firm enough for sexual activity<br>3 = firm enough for masturbation/foreplay<br>4 = firm enough for intercourse
ISUP grade group	x	x		1 = group 1 (gleason score 6)<br>2 = group 2 (gleason score 3+4=7)<br>3 = group 3 (gleason score 4+3=7)<br>4 = group 4 (gleason score 4+4=8)<br>5 = group 5 (gleason score 9-10)
Hormone therapy given to patients	x	x		1 = no hormone therapy given<br>2 = hormone therapy given

The presence of diabetes	x	x		1 = no diabetes<br>2 = diabetes
The four big treatments groups	x	x		1 = prostatectomy<br>2 = EBRT<br>3 = brachytherapy<br>4 = no active therapy
charlson comorbidity index simplified		x		0 = missing data<br>1 = no comorbidities<br>2 = 1 point<br>3 = >=2 points
abdominal/pelvic/rectal pain		x	6e	1 = no problem<br>2 = very small problem<br>3 = small problem<br>4 = moderate problem<br>5 = big problem
