# Supplementary material for: Development and external validation of multivariate prediction models for erectile dysfunction in men with localized prostate cancer
Source: PLoS One. 2023 Mar 3;18(3):e0276815. doi: 10.1371/journal.pone.0276815 (PMC9983834; doi:10.1371/journal.pone.0276815)
Supplement: S2 Table — (PDF) [file pone.0276815.s002.pdf]

Results of the statistical testing between all possible predictors and the erectile dysfunction outcome at 1-year post-diagnosis. These results include the p-value and the FDR-corrected q-values for each potential predictor.

| **Variable Name** | ***P*-value** | **Name of the statistical test** | **Significance** | **FDR-corrected q-value** | **Significance** |
|---|---|---|---|---|---|
| treatments | 3.73E-34 | Wilcoxon | * | 6.21E-33 | * |
| epic26_1_urineverlies1 | 4.15E-01 | Wilcoxon | | 2.45E-01 | |
| epic26_2_urineophouden1 | 5.68E-01 | Wilcoxon | | 3.07E-01 | |
| epic26_3_verbanden1 | 1.15E-01 | Wilcoxon | | 8.56E-02 | |
| epic26_4_nadruppelen1 | 1.44E-01 | Wilcoxon | | 1.02E-01 | |
| epic26_5_pijnplassen1 | 7.06E-01 | Wilcoxon | | 3.55E-01 | |
| epic26_6_bloedurine1 | 9.33E-01 | Wilcoxon | | 4.22E-01 | |
| epic26_7_zwakkestraal1 | 7.61E-02 | Wilcoxon | | 6.04E-02 | |
| epic26_8_aandrang1 | 2.99E-01 | Wilcoxon | | 1.89E-01 | |
| epic26_9_urineprobleem1 | 1.58E-01 | Wilcoxon | | 1.10E-01 | |
| epic26_10_drangontlasting1 | 6.62E-01 | Wilcoxon | | 3.41E-01 | |
| epic26_11_vakerontlasting1 | 6.08E-01 | Wilcoxon | | 3.22E-01 | |
| epic26_12_controledef1 | 7.60E-01 | Wilcoxon | | 3.72E-01 | |
| epic26_13_bloedontlasting1 | 5.27E-01 | Wilcoxon | | 2.92E-01 | |

| | | | | | |
|---|---|---|---|---|---|
| epic26_14_krampdarm1 | 9.49E-01 | Wilcoxon | | 4.26E-01 | |
| epic26_15_ontlastingprobleem1 | 7.50E-01 | Wilcoxon | | 3.69E-01 | |
| epic26_16_goederectie1 | 6.48E-22 | Wilcoxon | * | 2.16E-21 | * |
| epic26_17_goedklaarkomen1 | 1.45E-15 | Wilcoxon | * | 3.46E-15 | * |
| epic26_18_kwalerectie1 | 6.23E-24 | Wilcoxon | * | 5.19E-23 | * |
| epic26_19_kwanterectie1 | 2.88E-23 | Wilcoxon | * | 1.60E-22 | * |
| epic26_20_oordeelseksfunc1 | 3.13E-19 | Wilcoxon | * | 8.70E-19 | * |
| epic26_21_problseksfunc1 | 5.61E-02 | Wilcoxon | | 4.75E-02 | * |
| epic26_22_opvliegers1 | 5.74E-01 | Wilcoxon | | 3.10E-01 | |
| epic26_23_gevoeligeborsten1 | 6.40E-02 | Wilcoxon | | 5.29E-02 | |
| epic26_24_depressie1 | 2.14E-01 | Wilcoxon | | 1.43E-01 | |
| epic26_25_weinigenergie1 | 1.67E-02 | Wilcoxon | * | 1.63E-02 | * |
| epic26_26_gewicht1 | 1.29E-02 | Wilcoxon | * | 1.33E-02 | * |
| sCT | 6.60E-12 | Wilcoxon | * | 1.37E-11 | * |
| sCN | 9.89E-04 | Wilcoxon | * | 1.50E-03 | * |
| nLeeft | 6.82E-03 | Wilcoxon | * | 7.58E-03 | * |
| ch_indexgr | 3.01E-03 | Wilcoxon | * | 3.86E-03 | * |

| | | | | | |
|---|---|---|---|---|---|
| psa_diag | 5.91E-06 | Wilcoxon | * | 9.84E-06 | * |
| gleason_group | 1.18E-22 | Wilcoxon | * | 4.93E-22 | * |
| diabetes | 5.93E-03 | Wilcoxon | * | 6.80E-03 | * |
| cardiovascularDisease | 2.25E-03 | Wilcoxon | * | 3.06E-03 | * |
| hormoneTherapy | 1.74E-07 | Wilcoxon | * | 3.22E-07 | * |
| alg_rook | 8.18E-01 | Wilcoxon | | 3.90E-01 | |
| alg_alc | 6.54E-02 | Wilcoxon | | 5.37E-02 | |
