# Supplementary material for: Development and external validation of multivariate prediction models for erectile dysfunction in men with localized prostate cancer
Source: PLoS One. 2023 Mar 3;18(3):e0276815. doi: 10.1371/journal.pone.0276815 (PMC9983834; doi:10.1371/journal.pone.0276815)
Supplement: S3 Table — (PDF) [file pone.0276815.s003.pdf]

Results of the statistical testing between all possible predictors and the erectile dysfunction outcome at 2 years post-diagnosis. These results include the p-value and the FDR-corrected q-values for each potential predictor.

**Variable Name**	**P-value**	**Name of the statistical test**	**Significance**	**FDR-corrected q-value**	**Significance**
treatments	2.53E-17	Wilcoxon	*	9.19E-17	*
epic26_1_urineverlies1	1.39E-01	Wilcoxon		8.86E-02	
epic26_2_urineophouden1	9.30E-02	Wilcoxon		6.77E-02	
epic26_3_verbanden1	1.70E-01	Wilcoxon		1.01E-01	
epic26_4_nadruppelen1	1.04E-01	Wilcoxon		7.32E-02	
epic26_5_pijnplassen1	8.13E-01	Wilcoxon		3.35E-01	
epic26_6_bloedurine1	5.95E-01	Wilcoxon		2.69E-01	
epic26_7_zwakkestraal1	5.88E-02	Wilcoxon		4.79E-02	*
epic26_8_aandrang1	1.42E-01	Wilcoxon		8.96E-02	
epic26_9_urineprobleem1	9.17E-02	Wilcoxon		6.70E-02	
epic26_10_drangontlasting1	3.18E-01	Wilcoxon		1.66E-01	
epic26_11_vakerontlasting1	5.41E-01	Wilcoxon		2.51E-01	
epic26_12_controledef1	6.00E-01	Wilcoxon		2.71E-01	
epic26_13_bloedontlasting1	6.82E-01	Wilcoxon		2.97E-01	

epic26_14_krampdarm1	3.46E-01	Wilcoxon		1.77E-01	
epic26_15_ontlastingprobleem1	9.66E-01	Wilcoxon		3.75E-01	
epic26_16_goederectie1	2.01E-21	Wilcoxon	*	1.04E-20	*
epic26_17_goedklaarkomen1	2.86E-15	Wilcoxon	*	8.32E-15	*
epic26_18_kwalerectie1	4.23E-23	Wilcoxon	*	6.15E-22	*
epic26_19_kwanterectie1	2.21E-21	Wilcoxon	*	1.07E-20	*
epic26_20_oordeelseksfunc1	5.85E-15	Wilcoxon	*	1.42E-14	*
epic26_21_problseksfunc1	1.35E-03	Wilcoxon	*	1.61E-03	*
epic26_22_opvliegers1	8.15E-01	Wilcoxon		3.36E-01	
epic26_23_gevoeligeborsten1	3.57E-01	Wilcoxon		1.81E-01	
epic26_24_depressie1	2.99E-01	Wilcoxon		1.59E-01	
epic26_25_weinigenergie1	7.40E-01	Wilcoxon		3.15E-01	
epic26_26_gewicht1	1.78E-01	Wilcoxon		1.04E-01	
sCT	6.02E-09	Wilcoxon	*	1.09E-08	*
sCN	1.24E-02	Wilcoxon	*	1.20E-02	*
nLeeft	4.97E-06	Wilcoxon	*	8.03E-06	*
ch_indexgr	6.68E-05	Wilcoxon	*	8.83E-05	*

psa_diag	6.40E-05	Wilcoxon	*	8.54E-05	*
gleason_group	1.19E-13	Wilcoxon	*	2.48E-13	*
diabetes	3.20E-03	Wilcoxon	*	3.32E-03	*
cardiovascularDisease	2.85E-02	Wilcoxon	*	2.59E-02	*
hormoneTherapy	2.73E-03	Wilcoxon	*	2.93E-03	*
alg_rook	7.46E-01	Wilcoxon		3.17E-01	
alg_alc	1.21E-01	Wilcoxon		8.09E-02	
