# Supplementary material for: Development and external validation of multivariate prediction models for erectile dysfunction in men with localized prostate cancer
Source: PLoS One. 2023 Mar 3;18(3):e0276815. doi: 10.1371/journal.pone.0276815 (PMC9983834; doi:10.1371/journal.pone.0276815)
Supplement: S4 Table — These results include the p-value for each potential variable. (DOCX) [file pone.0276815.s004.docx]

Table 4. Results of the statistical testing between training and validation sets for important variables including demographics, predictors, and erectile dysfunction outcome in the 1-year dataset. These results include the p-value for each potential variable.

| **Variable Name** | **P-value** | **Name of the statistical test** | **Significance** |
| --- | --- | --- | --- |
| Treatments | 0.190115893227636 | Wilcoxon |  |
| Quality of erections | 0.546318927351216 | Wilcoxon |  |
| Frequency of erections | 0.940464447930849 | Wilcoxon |  |
| Lack of energy | 0.599693091099988 | Wilcoxon |  |
| Age | 0.781816183419378 | Wilcoxon |  |
| Gleason group | 0.256982343138352 | Wilcoxon |  |
| Diabetes | 0.183444519004922 | Wilcoxon |  |
| Cardiovascular Disease | 0.471059226178355 | Wilcoxon |  |
| Hormone Therapy | 0.321906885153677 | Wilcoxon |  |
| Alcohol use | 1.38717029768526e-05 | Wilcoxon | * |
| Outcome | 0.271840327146644 | Wilcoxon |  |
