# Supplementary material for: Development and external validation of multivariate prediction models for erectile dysfunction in men with localized prostate cancer
Source: PLoS One. 2023 Mar 3;18(3):e0276815. doi: 10.1371/journal.pone.0276815 (PMC9983834; doi:10.1371/journal.pone.0276815)
Supplement: S5 Table — These results include the p-value for each variable. (DOCX) [file pone.0276815.s005.docx]

Table 5. Results of the statistical testing between training and validation sets for important variables including demographics, predictors, and erectile dysfunction outcome in the 2-year dataset. These results include the p-value for each variable.

| **Variable Name** | **P-value** | **Name of the statistical test** | **Significance** |
| --- | --- | --- | --- |
| Treatments | 0.472862890917808 | Wilcoxon |  |
| Abdominal/pelvic/rectal pain | 0.846552111623635 | Wilcoxon |  |
| Quality of erections | 0.103465205946971 | Wilcoxon |  |
| Frequency of erections | 0.565355680054525 | Wilcoxon |  |
| Tumor T stage | 0.008418616187263 | Wilcoxon | * |
| Age | 0.0980238365756211 | Wilcoxon |  |
| Charlson comorbidity index simplified | 0.806889154826563 | Wilcoxon |  |
| Gleason_group | 0.79342315016652 | Wilcoxon |  |
| Diabetes | 0.0388195628164152 | Wilcoxon | * |
| Hormone Therapy | 0.310290588934216 | Wilcoxon |  |
| Outcome | 0.626260612025704 | Wilcoxon |  |
